# Restricted Dynamics and Para-Ortho Conversion of H_2_ Adsorbed in Micro- and Mesoporous Carbide-Derived Carbon: A Quasi- and Inelastic Neutron Scattering Study

**DOI:** 10.1021/acs.jpcc.4c08582

**Published:** 2025-02-25

**Authors:** Miriam Koppel, Rasmus Palm, Riinu Härmas, Mark T. F. Telling, Manh Duc Le, Tatiana Guidi, Kenneth Tuul, Maarja Paalo, Enn Lust

**Affiliations:** † Institute of Chemistry, University of Tartu, Ravila 14a, 50411 Tartu, Estonia; ‡ ISIS Neutron and Muon Facility, STFC Rutherford Appleton Laboratory, Chilton, Didcot OX11 0QX, U.K.; § School of Science and Technology, Physics Division, University of Camerino, I-62032 Camerino, Italy

## Abstract

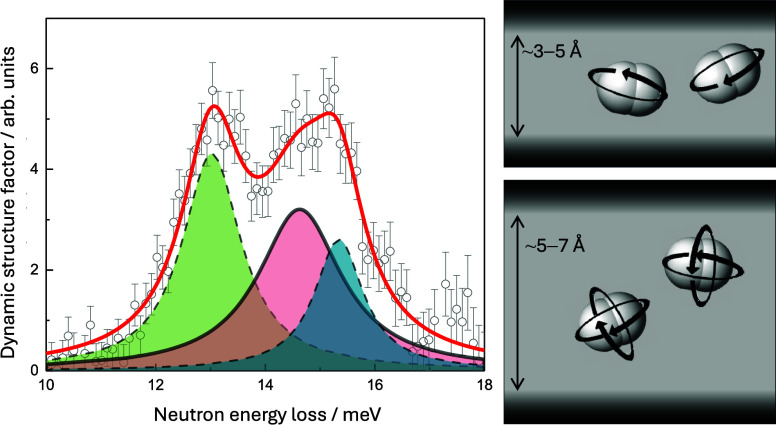

Strong confinement of hydrogen is important for adsorption-based hydrogen storage solutions, which are vital for the transition toward a hydrogen-based economy. The dynamics of hydrogen adsorbed in high-porosity TiC-derived carbon with relatively well-stacked graphenic layers for a carbide-derived carbon is investigated with *in situ* inelastic and quasi-elastic neutron scattering methods. Both the para-ortho rotational transition and elastic incoherent scattering factor are investigated. Hydrogen is translationally bound at temperatures of 20–80 K. At temperatures of 50 and 80 K, the adsorbed hydrogen exhibits localized jumps over 3.4 and 3.7 Å, respectively, along or between the ultramicropore walls. The restricted jumps of hydrogen in ultramicropores show the confining influence of specific adsorption sites present in the micropores of carbon materials, which limit hydrogen mobility and localize the hydrogen molecules within these pores. These findings yield new insights into the influence of hydrogen loading and temperature on the confinement of hydrogen and the development of carbonaceous adsorbents for high-density hydrogen storage.

## 1 Introduction

H_2_ storage is one of the most expensive components in achieving a H_2_-based economy considering production, storage, and transportation.^1^ One of the reasons for this high cost is the low volumetric density of H_2_. To increase the volumetric density, the contemporary industrial standard is to compress up to a high pressure of 300–700 bar or to liquefy at a low temperature of 20 K, where both methods involve considerable costs and energy losses related to cooling and compression.^2^ Physical adsorption of H_2_ in porous adsorbents is of interest for H_2_ storage because at the optimal temperatures and H_2_ pressures, 77 K and 100 bar, respectively, up to 30% more H_2_ is stored via cryo-adsorption compared to an adsorbent-free cryo-compression tank at equivalent conditions.^3^ Additional advantages of cryo-adsorptive storage in porous adsorbents are high reversibility and fast adsorption/desorption kinetics.^2^ H_2_ adsorbed in subnanometer pores can reach densities higher than that of liquid H_2_ (71 kg m^–3^), indicating that the adsorbed H_2_ is strongly confined.^4−6^ However, to reach a high H_2_ loading, a large total pore volume is required, which is usually achieved through the presence of larger than subnanometer pores. These larger pores strongly impact the H_2_ storage properties of adsorbents, i.e., H_2_ adsorption equilibrium, H_2_ self-diffusion rate, and mechanism of adsorption.^7−11^ Thus, further investigation is needed to understand the interplay of pore sizes on the confinement of H_2_ and, thus, the self-diffusion characteristics of adsorbed H_2_.

Different rotational states of H_2_ are of interest for investigating the confinement of H_2_ at different adsorption sites. H_2_ has two nuclear spin-alignment isomers, ortho-H_2_ (o-H_2_) and para-H_2_ (p-H_2_), where the nuclear spins are aligned parallel or antiparallel, respectively. o- and p-H_2_ isomers differ by the parity of quantum rotational numbers (*J*); p-H_2_ has even *J* values, while o-H_2_ has odd *J* values. The rotational energy levels are calculated as *E*_J_ = B *J* (*J* + 1), where B = 7.35 meV is the rotational constant of the H_2_ molecule.^12^ At room temperature, H_2_ is a 3:1 mixture of o- and p-H_2_, respectively. The thermodynamically stable ratio of p- and o-H_2_ isomers is dependent on temperature, i.e., at low temperatures (below 130 K), the para form becomes more prevalent. However, isolated p-H_2_ molecules cannot easily convert to o-H_2_ and vice versa since the natural, i.e., self-catalyzed H_2_ ortho-para (o → p) conversion is very slow and can take up to several days.^12^

In addition to the investigation of H_2_ confinement at different adsorption sites, the naturally occurring slow o → p conversion of H_2_ can be problematic for the storage of H_2_ in liquid form because the H_2_ o → p conversion is an exothermic process with a notable conversion enthalpy of −702 kJ kg^–1^ at 20 K.^13^ Therefore, the naturally occurring exothermic o → p conversion increases the temperature of stored liquid H_2_, causing H_2_ to continuously evaporate and, therefore, the internal pressure of the system to increase.^14^ Due to the o → p conversion, the liquid H_2_ needs regular cooling in order to stay in the liquid state. To avoid evaporation, H_2_ is converted to its 100% para form before liquefaction and before H_2_ enters the storage system with the use of catalysts.^15^ If the p → o conversion of H_2_ takes place in the storage system, the temperature of the system will be reduced as the p → o conversion is an endothermic process.^16^

In carbon materials, the paramagnetic sites on the edges of the graphenic planes, as well as some types of defects, have been shown to act as catalysts for the o → p transition.^17^ The disordered structure of porous graphenic carbons contains such catalytic sites to varying degrees depending on the exact hierarchical structure of the material. As a result, the porous structure of carbon adsorbents can be used to influence the rate of o → p conversion.^17^ For application as a cryo-adsorptive H_2_ storage media, identifying porous carbon materials and their porous structures that are able to stabilize the o-H_2_ form is of interest. Such materials would avoid the energy release from o → p conversion and, thus, yield a potentially more energy-efficient H_2_ cryostorage system.

### 1.1 Rotational Transitions of H_2_ Adsorbed in Various Pores

In addition to the potential practical interest in investigating the o → p transition of H_2_ in porous materials, the o → p transition can also yield important insight into the mechanism of H_2_ adsorption. The rotational energy of a H_2_ molecule (14.7 meV) is comparable to the energy of an adsorbed H_2_ molecule (∼30 meV per molecule).^17,18^ Therefore, the H_2_ p → o conversion energy can be influenced by adsorption and the differences in the conversion energy can be used to investigate the properties of the adsorption sites. For example, a H_2_ p → o transition at 14.7 meV indicates the presence of adsorbed H_2_ molecules which are translationally bound but free to rotate in any direction. The rotational energy of a H_2_ molecule has been shown to shift from 14.7 meV toward 14.5 meV if adjacent H_2_ molecules are hindering each other’s rotations and altering the transition energies.^6,18−22^ Thus, analyzing the position of the p → o transition band yields information about the H_2_–H_2_ and H_2_-adsorbent interactions.^6,9,17−22^

The interactions of o- and p-H_2_ with the porous structure are different. This is caused by the difference in the anisotropic component of the adsorption potential of o- and p-H_2_. Where the physisorption energy is a sum of the isotropic and anisotropic components of the adsorption potential and where the isotropic component is equal for both o- and p-H_2_. The energy of the o-H_2_ rotational levels can change because of the anisotropic component, while the energy of the p-H rotational level is unchanged. Therefore, due to the decrease in the o-H_2_ energy level in specific anisotropic adsorption sites, o-H_2_ can preferably adsorb in these adsorption sites compared to p-H_2_.^18^

If the H_2_ molecule is translationally bound by the adsorption site, but still free to rotate in any direction, the adsorption site is most likely in supermicro- and/or mesopores,^23^ i.e., pores with widths 7 Å < *w* < 20 and 20 Å < *w* < 500 Å, respectively. The potential energy from the pore walls, which is known as the adsorption potential of the pore walls, does not overlap in the case of the supermicro- and mesopores, and the adsorption of H_2_ is energetically similar to the adsorption at an independent surface.^24^ Therefore, the energetic environment at these adsorption sites is isotropic and allows the H_2_ molecules to rotate in any direction with almost no preference.

If the width of the pore is below 7 Å (ultramicropores),^23^ the adsorption potentials of opposing pore walls start to overlap, and the rotations of H_2_ are no longer equivalent in all directions at the resulting adsorption sites, i.e., the adsorption potential is anisotropic and H_2_ can be considered a hindered rotor, and the asymmetricity and splitting of the H_2_ p → o transition band have been shown to be characteristic to such a case.^6,17,19−21^ The rotational motions of H_2_ molecules tend to be confined favorably either along the surface-normal or -parallel direction in the case of adsorption in sites with high anisotropy and potential.^19,20^ Thus, the splitting of the adsorbed H_2_ molecule rotational sublevels is caused by the surface-normal aligned H_2_ rotations or the surface parallel aligned H_2_ rotations exhibiting different energy compared to the free rotation of the H_2_ molecule.^19,20^ The energy difference of the two split bands and the ratio of their areas yields information about the degree of anisotropy experienced by the H_2_ molecule and, thus, the preferred orientation of the H_2_ molecular axis.^19,20^

Therefore, the preferred orientation of H_2_ molecules at the surface depend on the effective width of the pore. The highly anisotropic adsorption potential, i.e., adsorption is not equally probable in all directions on the surface, of ultramicropores favors the adsorption of H_2_ molecules with the molecular axis of H_2_ parallel to the pore wall. This is generally more stable and, thus, favored due to the ellipsoidal shape of the H_2_ molecule electronic structure.^25,26^

### 1.2 Carbide-Derived Carbons as Model Materials

Carbide-derived carbons (CDCs) are well-suited model materials for investigating the confinement of H_2_ in its high-energy adsorption sites since the structure of CDCs can be influenced by precursor carbide chemical composition and structure as well as synthesis conditions such as temperature.^27−31^ CDCs can be efficiently used in many applications, for example as electrode materials for supercapacitors, batteries, and polymer electrolyte fuel cells and adsorbents for H_2_ and other gas storage.^32−37^ CDCs have a relatively versatile structure, e.g., their porous structure can vary from mainly ultramicroporous (close to 100%) to predominantly mesoporous, where 78% of the porous structure is made up of mesopores by volume.^38,39^

### 1.3 *In Situ* Neutron Scattering for Analysis of H_2_ Diffusion

Neutron scattering is a well-suited technique for investigating the dynamics and properties of adsorbed H_2_ because, of all periodic elements, H_2_ has the largest incoherent neutron scattering cross sections (80.26 × 10^–28^ m^2^ or 80.26 barn at 25 meV) and all incoherent scattering yields information about the self-motion of a H_2_ molecule.^40−42^ In addition, being a neutral particle, neutrons have a deep penetration depth, enabling *in situ* measurements to be performed using complex thick-walled sample environments.^40,41^ Quasi-elastic neutron scattering (QENS) can be applied to study the self-diffusion of H_2_ adsorbed in various adsorbents, e.g., zeolites, micro- and mesoporous carbons, and carbon aerogels, as the used incident neutron energies (∼2 meV) enable to probe the self-diffusion of adsorbed H_2_ at time scales characteristic to the processes.^9,10,43−47^ However, the free self-diffusion of the H_2_ molecule can be hindered by strong H_2_-adsorbent interaction at a high-energy adsorption site.^6,9,17,19,20^ Here, the energy exchange between the neutron and the H_2_ molecule does not appear in the quasi-elastic regime. Instead, the strongly adsorbed H_2_ molecule exhibits a well-defined rotational energy level transition (at 14.7 meV), which is detected in the inelastic neutron scattering (INS) region.^6,9,17,19,20^ This transition has been shown to be related to the adsorption mechanism of H_2_ and, thus, the INS technique yields invaluable information about the effect of different porous structures on the properties of strongly confined H_2_, i.e., effectively immobile H_2_ at high-energy adsorption sites.^6,9,17,19,20^

### 1.4 Motivation

Carbide-derived carbon adsorbent synthesized from TiC, synthesized with the sol–gel method and henceforth named sol–gel TiC-CDC, is used to investigate effect of hierarchical micro/mesoporous structure, high surface area, and overall versatile properties the sol–gel TiC-CDC on the self-diffusion and confinement of H_2_.^8,48^ The high ratio of ultramicro-, micro-, and mesopores, the relatively well-ordered graphenic regions, with some defects in the graphenic layers, make sol–gel TiC-CDC a unique adsorbent to study the dynamics of adsorbed H_2_.^8^ As such, in our previous study with the same sol–gel TiC-CDC and using the QENS analysis we determined that the self-diffusion of H_2_ adsorbed is rotational in the ultramicropores, translational in micro- and mesopores, and the H_2_ self-diffusion phenomena takes place over multiple time scales.^8^ The hierarchical pore structure, chemical purity, and suitable carbon structure make sol–gel TiC-CDC a highly promising carbon material for a wide array of applications, for example as a supercapacitor electrode material.^49^

As a continuation of the investigation performed in ref (8), herein the focus is on the characterization of highly confined H_2_ adsorbed in sol–gel TiC-CDC through the analysis of p → o conversion and EISF. Thus, *in situ* INS and QENS methods are used in the temperature range from 20 to 100 K and at surface coverages from 30 to 294%, at 20 K. These conditions correspond to the temperature range of interest for practical cryo-adsorptive H_2_ storage and other applications. At the lowest applied surface coverage, 30% at 20 K, where H_2_ is preferably adsorbed in high-energy adsorption sites in the smallest pores, is investigated. The higher surface coverages −133 and 294% at 20 K, are applied to investigate the combined effect of strong and medium adsorption sites, present in larger pores, to the confinement of H_2_.

## 2 Methods

### 2.1 Material Synthesis and Physical Characterization

Material synthesis and characterization methods have been described in detail in refs (8,39), respectively, and are briefly summarized below.

The sol–gel synthesis method was used to prepare the TiC precursor used for the carbide-derived carbon (CDC). The CDC material used in this study (from here on referred to as sol–gel TiC-CDC) was synthesized by chlorination of the precursor sol–gel TiC. The synthesis process is described in detail in ref (39).^39^

2D-NLDFT-HS model^50−52^ was used to globally fit the measured N_2_, CO_2_, and H_2_ gas adsorption isotherm data to calculate the pore size distribution and the specific surface area, *S*_DFT_, and pore volume, *V*_DFT_.

The first-order Raman spectra of the sol–gel TiC-CDC was obtained at an excitation wavelength of 514 nm and was fitted with 4 Lorentzian and 1 Gaussian peak functions to separate the D_S_, D_A_, G_S_, G_A_, and D′ bands (L + L + G + L + L, respectively). The applied Raman spectra deconvolution procedure has been shown to be suitable for CDCs.^48^ The integrated intensity ratio of the D- and G-bands, *I*_∑D_/*I*_∑G_, and the full width at half maximum of the D-band, FWHM_DA_, were obtained for characterizing defects in the graphitic structure of the sol–gel TiC-CDC.^48,53−55^

The wide-angle X-ray scattering (WAXS) curve was obtained with Cu Kα radiation. The WAXS curve was fitted with the CarbX software^56,57^ to obtain the average graphene-like platelet size, *L*_a_, the average stacking size, *L*_c_, the average interlayer spacing, *a*_3_, and the average number of graphene-like layers per stack, ⟨*N*⟩.

### 2.2 Neutron Scattering

Neutron scattering experiments were performed using the MARI^58^ and IRIS^59^ spectrometers at the ISIS Pulsed Neutron and Muon Source, UK, and the neutron scattering data can be accessed at refs (60,61). As the first step, before the neutron scattering measurements the sol–gel TiC-CDC sample was outgassed at 300 °C. After that, the sample was transferred to an annular cylindrical aluminum sample can. To ensure that beam transmission would be more than 90%, a sample thickness of 0.2 cm was chosen. At this level, multiple scattering effects can be considered negligible. The measurement plan and the instrument parameters used are described in ref (8) in greater detail.

As a summary of the most important aspects of the neutron scattering experiments, the sample was cooled using a He close-cycle refrigerator and the gas handling system for dosing H_2_ can be seen in SI (Supporting Information) Figure S1 in section 1. As the first step, the signal from the outgassed sol–gel TiC-CDC was measured in the temperature range 20–100 K. Next, the sample was cooled to 77 K, and the closed sample cell was dosed with H_2_ for three total H_2_ loadings per 1 g of sol–gel TiC-CDC (*n*_H2_) −1.7, 10, and 31 mmol g^–1^. The adsorption equilibrium was established over approximately 1 h and after that the sample cell was hermetically sealed, isolating it from the gas handling system. These *n*_H2_ values were chosen to ensure surface coverage values of 30, 133, and 294% at 20 K for both experiments conducted on MARI and IRIS. A surface coverage value greater than 100% indicates that a full monolayer occupation with H_2_ has been achieved and that some of the H_2_ is adsorbed in subsequent layers farther from the pore walls. The exact H_2_ loading pressures and calculations are in SI section 1.

The neutron scattering data from sol–gel TiC-CDC and the adsorbed H_2_ was collected in the temperature range from 10 to 100 K. The data obtained at the lowest temperature of 10 and 20 K on IRIS and MARI spectrometers, respectively, and at *n*_H2_ = 1.7 mmol g^–1^, was used for instrument calibration and as an estimate of the instrument resolution function during subsequent analysis. At these lowest applied *T* and *n*_H2_ conditions, the H_2_ adsorbed in the sol–gel TiC-CDC is assumed to be immobile on the experimental time scales accessible by the used neutron instruments and experimental setups. The surface coverages and pore volume occupancies of H_2_ in sol–gel TiC-CDC are calculated at all experimental conditions for both experiments and are presented in SI section 1. The data reduction and analysis for both experiments were carried out using the Mantid software package^62^ and OriginPro (2016) software.

#### 2.2.1 Inelastic Neutron Scattering

The MARI spectrometer was configured with the Fermi chopper running at 400 Hz and phased to pass neutrons with incident energies around 20 meV, which enabled the detection of neutrons in the energy transfer range ±20 meV. To analyze the inelastic region of the spectra, i.e., around 14.7 meV, the reduced data was summed over all scattering vector, *Q*, values into one data set. The quasi-elastic region (centered around the elastic peak and extending up to ±6 meV) of the MARI spectra has been analyzed and published previously in ref (8).^8^

For the analysis of processes in the inelastic region, the H_2_ para to ortho (p → o) transition band is fitted with three Lorentzian functions. The H_2_ p → o transition has been shown to split into three components and the ratio of the lowest and highest energy components is ∼2:1.^6,18,20,63^ Therefore, the intensity ratio of the lowest and highest energy bands was fixed at ∼2:1 throughout the fitting. The fitting results are shown in detail in SI section 2.

The inelastic neutron scattering data was measured and fitted at all applied *n*_H2_ values and at fixed *T* values of 20, 40, 60, and 80 K.

#### 2.2.2 Quasi-elastic Neutron Scattering

The IRIS spectrometer was configured to analyze the energy of the scattered neutron beam using the 002 graphite (PG002) analyzer reflection. The setup used enabled scattering events in the energy transfer range −0.3 to 1.2 meV to be detected. The quasi-elastic region of the spectra was analyzed with the reduced data being collated into five distinct *Q* groups in the *Q*-range 0.42–1.8 Å^–1^. The quasi-elastic region of the spectra at *n*_H2_ ≥ 10 mmol g^–1^ has been analyzed and published previously in ref (8).

To the quasi-elastic region, the incoherent scattering function, *S*(*Q*,*E*), was fitted at *n*_H2_ = 1.7 mmol g^–1^ and at *T* values of 30, 50, and 80 K. The *S*(*Q*,*E*) comprised of elastic and quasi-elastic scattering components, an instrumental energy resolution function, and a background (eq 1)
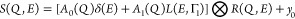
1Here, *S*(*Q*,*E*) is the incoherent scattering function, *Q* is the scattering vector, *E* is the energy transfer, δ(*E*) is the Dirac delta function describing the elastic scattering intensity, *A*_0_ and *A*_1_ are the fractions of elastic and quasi-elastic scattering signals, respectively, *L* is the Lorentzian function describing the quasi-elastic component, Γ is the half-width at half maximum (HWHM) of the quasi-elastic component, *R*(*Q*,*E*) in the instrumental resolution function, and *y*_0_ is the background.

Modeling the associated Elastic Incoherent Structure Factor (EISF), *A*_0_ (eq 2), yields information about the geometry of the motion and, possibly, the percentage of mobile species contributing to the QENS signal.
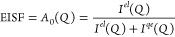
2The ratio of EISF from the mobile fraction of species can depend on temperature. Consequently, not the theoretical EISF (*A*_0_(*Q*)), but the effective EISF (*A*_0_′(*Q*)), which accounts for the presence of mobile and immobile particles in the system is used. The effective EISF can be calculated from the theoretical EISF as *A*_0_′(*Q*) = *p*_s_ + *p*_m_*A*_0_(*Q*), where *p*_s_ and *p*_m_ are the relative fractions of static (over the experimental time scale) and mobile species, respectively, and which sum up to unity.

The motions are analyzed by fitting experimentally determined EISF responses to theoretical predictions. Three different EISF models were applied:

1. Rotational jumps between two equidistant sites on a circle with a diameter *d*.^41^
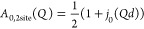
3where *j*_0_ is the zero-order spherical Bessel function of the first kind.
2. Continuous rotational diffusion on the surface of a sphere with a diameter *d*.^41^
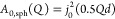
4
3. Continuous rotational diffusion within the volume of a sphere with a diameter *d*.^41^
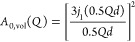
5where *j*_1_ is the first-order spherical Bessel function of the first kind.

The description of the fitting procedure and the analysis of QENS and EISF fittings are given in SI section 3.

## 3 Results

### 3.1 Physical Characterization

The physical characterization of the same sol–gel TiC-CDC material has previously been published in ref (8) and the results are briefly summarized in this section.

**Figure 1:**
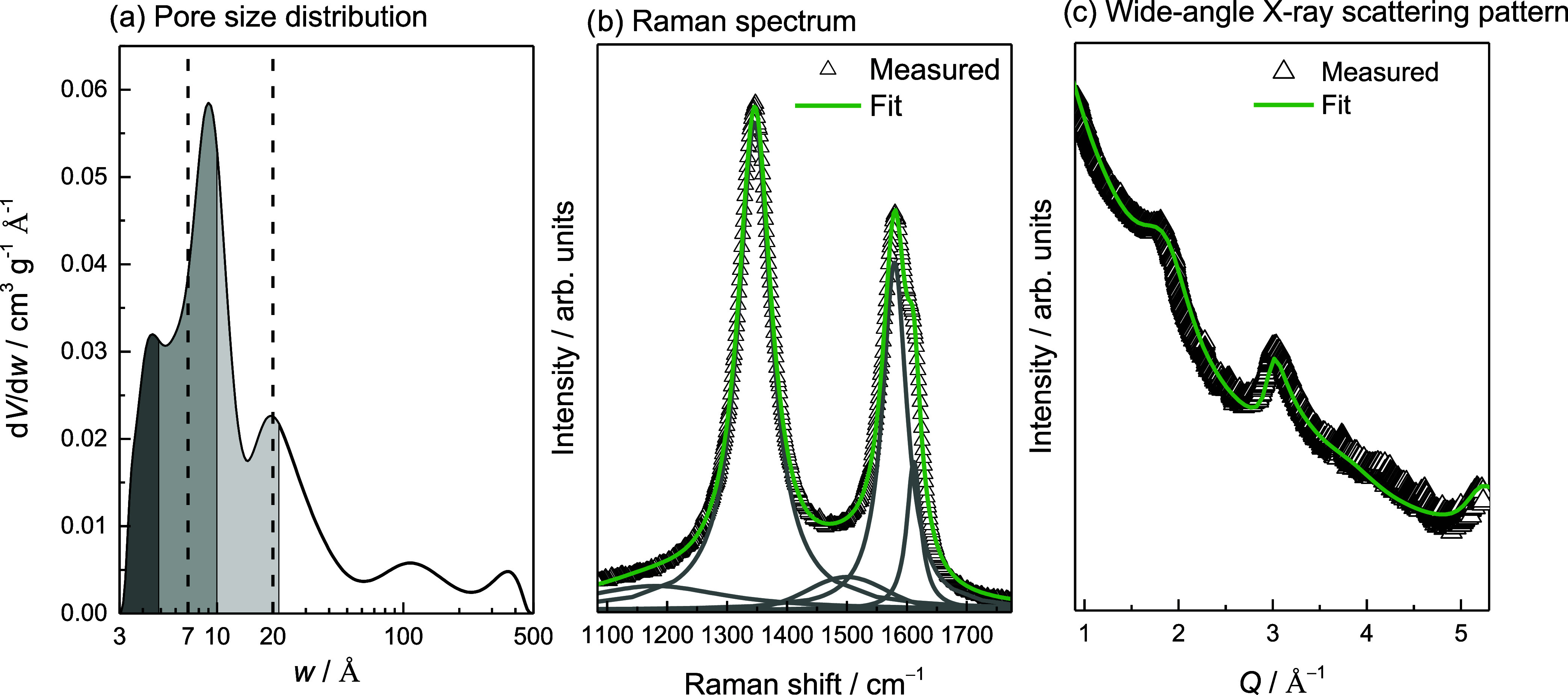
(a) Pore size distribution of sol–gel TiC-CDC, where the vertical dashed lines at 7 and 20 Å denote the upper limits of ultramicro- and micropores, respectively. The dark, medium, and light gray filled areas denote the volume of occupied pores at 20 K and at *n*_H2_ = 1.7 mmol g^–1^, *n*_H2_ = 10 mmol g^–1^, and *n*_H2_ = 31 mmol g^–1^, respectively, assuming that smaller pores are filled first and the density of adsorbed H_2_ is equal to that of liquid H_2_. (b) The deconvolution of the first-order Raman spectrum of sol–gel TiC-CDC. The bands from left to right are denoted as D_S_, D_A_, G_S_, G_A_, and D′. (c) Wide-angle X-ray scattering data of sol–gel TiC-CDC and fit obtained with the CarbX software. The scattering vector modulus, *Q*, is defined as *Q* = 4π sin(θ)/λ, where 2θ is the scattering angle. Reproduced from ref (8). Available under a CC-BY 4.0 license. Copyright 2024 M. Koppel.

The pore size distribution of sol–gel TiC-CDC calculated by globally fitting N_2_, CO_2_, and H_2_ adsorption isotherms can be seen in Figure 1a. Sol–gel TiC-CDC exhibits *S*_DFT_ value of 1560 m^2^ g^–1^ and *V*_DFT_ value of 2.40 cm^3^ g^–1^, where ultramicropores make up 5%, supermicropores make up 17%, and mesopores make up 78% of the total determined pore volume (pore width, *w*, from 3 to500 Å).^8^

At 20 K and at *n*_H2_ = 1.7 mmol g^–1^, ∼2% of the total pore volume is occupied with H_2_, assuming that the density of adsorbed H_2_ is equal to that of liquid H_2_ (Table 1, calculations in SI section 1). As ultramicropores make up 5% of the total pore volume and the smallest pores are filled first, at *n*_H2_ = 1.7 mmol g^–1^, H_2_ mostly occupies the strongest adsorption sites in ultramicropores.

**Table 1:** H_2_ Loading (*n*_H2_) at 77 K, Pore Volume Occupancy, and Surface Coverage at 20 K

H_2_ loading (*n*_H2_)/mmol g^–1^	1.7	10	31
pore volume occupancy/%	2	11	24
surface coverage/%	30	133	294

At 20 K and at *n*_H2_ = 10 and 31 mmol g^–1^, 11 and 24% of the total pore volume is occupied with H_2_, respectively (Table 1, calculations in SI section 1). Micropores make up 22% of the total pore volume, meaning that at *n*_H2_ = 10 mmol g^–1^, H_2_ occupies most of the ultramicropores and some of the supermicropores. At *n*_H2_ = 31 mmol g^–1^, H_2_ occupies all the micropores and some of the mesopores. The surface coverage calculations suggest that at 20 K and at *n*_H2_ = 10 and 31 mmol g^–1^, 133 and 294% of the monolayer is filled, respectively (Table 1, calculation in SI section 1). Thus, suggesting that at these conditions, some of the adsorbed H_2_ is in subsequent layers in addition to the H_2_ monolayer and a complete fillment on micropores (Figure 1a) or partial fillment of micropores with H_2_ adsorbed also in mesopores is achieved.

Raman spectroscopy and wide-angle X-ray scattering (WAXS) fitting results can be seen in Figure 1b,c, respectively. The main parameters obtained from the deconvolution of Raman spectrum and fitting of WAXS pattern are brought in Table 2.

**Table 2:** Main Parameters Obtained from the Deconvolution of Raman Spectra and Fitting of WAXS Patterns of Sol-Gel TiC-CDC from ref (8)^a^

*I*_ΣD_/*I*_ΣG_	FWHM_DA_ / cm^–1^	*L*_a_ / Å	*L*_c_ / Å	*a*_3_ / Å	⟨*N*⟩
1.59 ± 0.02	67.4 ± 0.2	50 ± 15	12.5 ± 2.3	3.4 ± 0.2	2.76 ± 0.55

a *I*_ΣD_/*I*_ΣG_, integrated intensity ratio of D- and G-bands from Raman spectrum; FWHM_DA_, full width at half maximum of the deconvoluted D_A_ band of the Raman spectrum; *L*_*a*_, average graphene-like platelet size from WAXS; *L*_c_, average stacking size from WAXS; *a*_3_, average interlayer spacing from WAXS; and ⟨*N*⟩, the average number of graphene-like layers per stack from WAXS.

### 3.2 Inelastic Neutron Scattering

**Figure 2:**
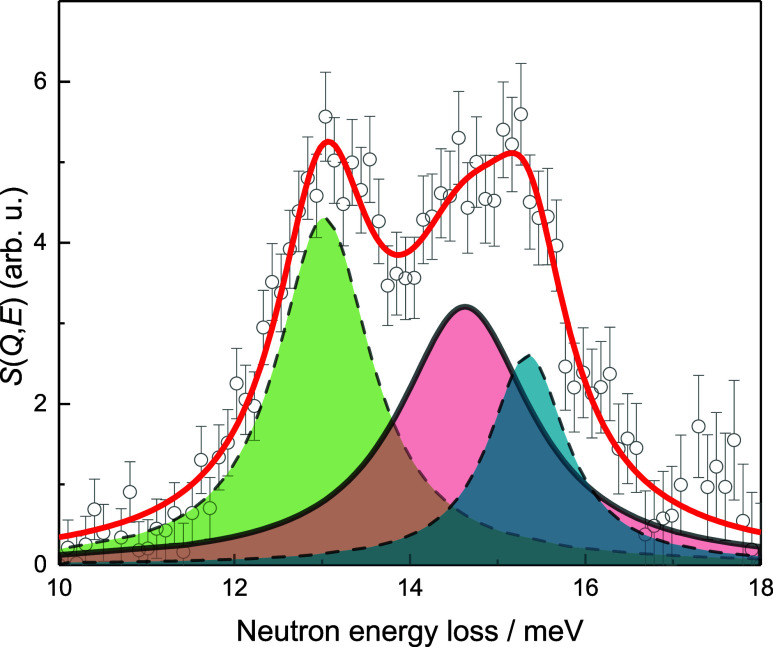
H_2_ para-ortho (p → o, *J* = 0 → 1) transition band at *T* = 20 K and *n*_H2_ = 1.7 mmol g^–1^ fitted with three Lorentzian functions (red solid line denoting the cumulative fit).

At around Δ*E* = 10–18 meV, at all *n*_H2_ values, and in the temperature range of 20–80 K, a broad asymmetric peak is observed (Figure 2). This broad feature is attributed to the para-ortho (p → o, *J* = 0 → 1) transition of H_2_ adsorbed in sol–gel TiC-CDC. This p → o transition has been predicted computationally, and observed experimentally, for H_2_ adsorbed in different adsorbents, e.g., activated carbons, carbon nanotubes, carbon nanohorns, and ultramicroporous carbons.^17,19−21,63^ H_2_ p → o transition bands with different shapes and intensities have been observed in the temperature range of 1.5–77 K and at various H_2_ loadings in these studies.

The measured p → o transition peak is deconvoluted into three main components (Figure 2) by fitting with three Lorentzian functions at all measured conditions. The main obtained parameters can be seen in Table 3 and the rest of the fitting results in Figure S3 and Table S6. At lower temperatures of ≤80 K and at lowest *n*_H2_ = 1.7 mmol g^–1^, the shape of the p → o transition band is asymmetric and the band is strongly split (Figures 2 and S3). The energy difference of the split ortho bands (Δ*E*) becomes generally greater with the increase in temperature and the total integrated area of the p → o transition band (*A*_tot_) decreases with increased temperature (Table 3). In addition, with increased *n*_H2_, both the proportion of the free-rotation band (*A*_free_/*A*_tot_) (Figure 2, red shaded area) and the *A*_tot_ increase (Table 3).

**Table 3:** Fitting Results and Calculated Parameters of H_2_ p → o Transition Bands^a^

*n*_H2_ / mmol g^–1^	*T* / K	*E*_free_ / meV	*A*_tot_ / arb. units	*A*_free_/*A*_tot_ / %	Δ*E* / meV
1.7	20	14.63 ± 0.33	23 ± 9	41 ± 21	2.32 ± 0.15
	40	14.70 ± 0.10	16 ± 5	24 ± 17	2.24 ± 0.34
	60	14.84 ± 0.08	10 ± 2	31 ± 11	3.21 ± 0.22
	80	14.81 ± 0.14	4 ± 1	41 ± 18	2.94 ± 0.26
10	20	14.51 ± 0.04	75 ± 16	57 ± 10	1.99 ± 0.16
	40	14.61 ± 0.11	66 ± 27	59 ± 20	2.63 ± 0.39
	60	14.79 ± 0.19	42 ± 32	51 ± 38	3.00 ± 1.00
	80	14.61 ± 0.22	18 ± 7	48 ± 20	3.68 ± 0.45
31	20	14.47 ± 0.03	305 ± 46	71 ± 8	2.48 ± 0.82
	40	14.54 ± 0.06	161 ± 43	68 ± 13	2.64 ± 0.36
	60	14.70 ± 0.34	91 ± 26	47 ± 14	3.32 ± 0.74
	80	14.87 ± 0.13	43 ± 13	53 ± 16	4.08 ± 0.26

a *E*_free_, free-rotation band energy position; *A*_tot_, total area of the band; *A*_free_/*A*_tot_, free-rotation band area relative to the total area of the band; Δ*E*, energy difference of the split ortho bands.

At *T* = 20 K and at all *n*_H2_ values, the *E*_free_ value is at a lower energy than 14.7 meV (Table 3), where 14.7 meV is characteristic for solid H_2_.^64^ The shift of the *E*_free_ value is most evident at *T* = 20 K and under the highest applied loading, *n*_H2_ = 31 mmol g^–1^. At these conditions, the highest surface coverage value of 294% (Table 1) is achieved in the experiment. With the increase in temperature above 20 K, the *E*_free_ value shifts from near 14.5 meV to around 14.7 meV (Table 3). Also, the *A*_free_/*A*_tot_ value most likely has a decreasing tendency with increasing temperature (Table 3), which is only reliably evident at the highest applied *n*_H2_ value because of the high fitting errors resulting from the p → o transition devonvolution with three Lorentzian functions (Figure S3).

No H_2_ o → p conversion is detected (signal measured over 3 h and averaged) for H_2_ adsorbed in sol–gel TiC-CDC under all applied temperature and H_2_ loading conditions. The lowest applied H_2_ loading and temperature were *n*_H2_ = 1.7 mmol g^–1^ and 20 K, respectively, in the measured energy transfer range −20 to 20 meV (SI section 4).

### 3.3 Quasi-elastic Neutron Scattering

**Figure 3:**
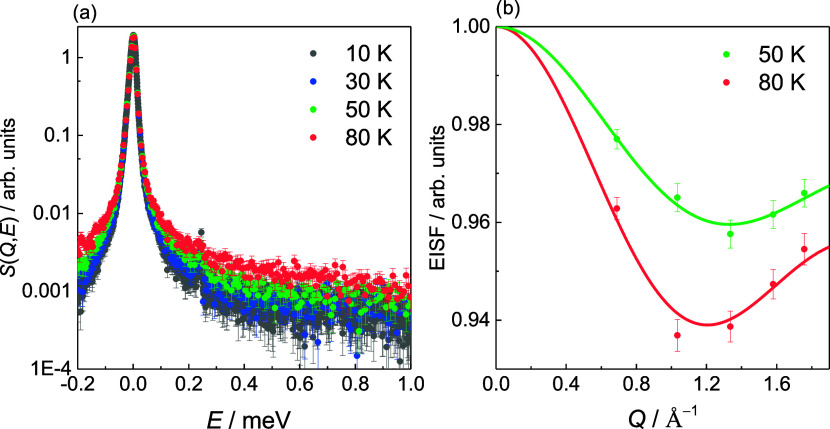
(a) *S*(*Q*,*E*) (summed over *Q*) of adsorbed H_2_ from 10 K (used as resolution function) to 80 K (shown in the figure) and (b) the elastic incoherent structure factor (EISF) response at 50 and 80 K at *n*_H2_ = 1.7 mmol g^–1^ fitted with the model assuming jumps between two equidistant sites on a circle (solid line). Reproduced from ref (8). Available under a CC-BY 4.0 license. Copyright 2024 M. Koppel.

Quasi-elastic broadening of the *S*(*Q*,*E*) is investigated at *n*_H2_ = 1.7 mmol g^–1^ and *T* values of 30–80 K. At these conditions, the para form of H_2_ adsorbed in sol–gel TiC-CDC should be prevalent since it is thermodynamically more stable. While 99.8% of H_2_ is in its para form at equilibrium at 20 K, this percentage decreases to ∼50% as the temperature increases to 77 K. However, the neutron scattering cross-section of p-H_2_ (0.6 × 10^–28^ m^2^ at 1 meV) is negligible compared to that of o-H_2_ (95 × 10^–28^ m^2^ at 1 meV).^42^ Therefore, the self-diffusion of p-H_2_ is considerably more difficult to detect with neutrons compared to the self-diffusion of o-H_2_. Thus, the possibility of detecting and analyzing the quasi-elastic broadening is an indication of the presence of o-H_2_ adsorbed in sol–gel TiC-CDC at these conditions. Broadening of *S*(*Q*,*E*) (Figure 3a) indicates that some of the H_2_ adsorbed in sol–gel TiC-CDC can self-diffuse at 30–80 K. The line width analysis from ref (8) indicates that the self-diffusion mechanism of H_2_ adsorbed in the high-energy adsorption sites is rotational, localized, and restricted.

The *Q*-dependence of the elastic incoherent structure factor (EISF) at *n*_H2_ = 1.7 mmol g^–1^ indicates that the rotational motions of self-diffusive H_2_ are geometrically constrained (Figure 3b). To determine the geometry of the rotational motions, the EISF response shown was fitted using three distinct models: (1) H_2_ performing rotational jumps between two equidistant sites on a circle (eq 3), (2) continuous rotational diffusion of H_2_ on the surface of a sphere (eq 4), and (3) continuous rotational diffusion of H_2_ within the volume of a sphere (eq 5) (SI section 3). The *d* value is determined from the first minimum of the EISF vs *Q* graph (Figure 3b), which is reliably observed in the available *Q*-range. Even though the exact geometry of motion is hard to determine, based on the fitted rotational jump models (Figure S7), the rotational jumps between two equidistant sites on a circle is most likely to characterize the obtained EISF data. Thus, the results obtained based on the rotational jumps between two equidistant sites are used henceforth. The obtained *d* values, i.e., average jump distances between two sites, are 3.4 ± 0.1 and 3.7 ± 0.1 Å at 50 and 80 K, respectively.

## 4 Discussion

Sol–gel TiC-CDC adsorbent belongs to a larger group of CDCs inside which its’ structural properties have been compared.^8,48^ Based on WAXS analysis, the average stacking size of graphene-like layers, *L*_c_, in sol–gel TiC-CDC is 12.5 Å, which is larger compared to other CDCs. The average graphene-like platelet size, *L*_a_, is in a similar range for all CDCs (50 Å) (Table 1), indicating a comparable level of ordered graphene-like platelet sizes for the compared CDCs. In addition, the average number of stacked graphenic layers, ⟨*N*⟩, and the average interlayer spacing between graphenic layers, *a*_3_, differentiate most remarkably between sol–gel TiC-CDC and the other 32 CDCs investigated in ref (48). The sol–gel TiC-CDC has on average 2.76 graphenic layers per stack, while other CDCs have on average ≤1 layer per stack, and the *a*_3_ value of sol–gel TiC-CDC is lower than that for the other 32 CDCs investigated in ref.^48^ Thus, indicating a greater graphenic interlayer ordering in sol–gel TiC-CDC compared to the set of investigated CDCs. This is also supported by the Raman analysis as the parameter *I*_∑D_/*I*_∑G_ (Table 1) exhibits similar values to other CDCs.^48^ The lower FWHM_DA_ value is characteristic of CDC with a larger coherent graphene-like platelet domain size.^48^

Thus, the sol–gel TiC-CDC exhibits well-ordered domains of graphene-like platelets compared to 32 other porous CDCs (Figure 4 from ref (48)) and serves as an interesting model carbon material that simultaneously exhibits a high porosity with a hierarchical wide pore size distribution and a considerable amount of graphene platelets with low amount of defects. Such structure has been shown to provide high-energy adsorption sites for adsorbed H_2_ as well as paramagnetic sites to catalyze the p → o conversion of adsorbed H_2_ molecules.^17,18^

The first p → o transition (*J* = 0 → 1) is observed for H_2_ adsorbed in sol–gel TiC-CDC at ∼14.7 meV (Figure 2, red shaded area). This p → o transition is assigned to the p-H_2_ molecules adsorbed in sol–gel TiC-CDC that are translationally bound (i.e., solid-like) but free to rotate in any direction, i.e., free-rotation band as in ref (6). The freely rotating H_2_ molecules are adsorbed at adsorption sites where the potential of opposing pore walls overlap and create a confining environment for the H_2_ molecule. However, the effective width of the pore is large enough to leave room for the H_2_ molecule to rotate freely, i.e., in larger ultramicropores with *w* ∼ 5–7 Å. Such pores can accommodate two layers of adsorbed H_2_.^65^ The p → o transition (*J* = 0 → 1) has been also observed at *E*_J=0→1_ = 14.7 meV for pure solid H_2_ at 4 K which behaves as a free rotor.^64^

The asymmetric shape and splitting of the H_2_ p → o transition band is caused by the splitting of H_2_ rotational sublevels (Figure 2).^6,17,20,21^ This indicates the presence of H_2_ molecules that are adsorbed in high-energy adsorption sites with high anisotropic potential in which rotational motions in specific directions are strongly hindered, i.e., in smaller ultramicropores with *w* ∼ 3–5 Å. In such pores, the adsorption potentials of opposing pore walls overlap and only one layer of H_2_ can be accommodated.^24,25^ Therefore, the adsorbing molecule will interact with both opposing pore walls through van der Waals forces and, thus, is strongly adsorbed. It should be stressed that adsorption sites in ultramicropores can be highly anisotropic, e.g., in the case of defects in the carbon structures. At these sites, the rotations of H_2_ will not be equally favorable in every direction, which causes the o-H_2_ rotation levels to split.^6,20^

Generally, the asymmetric shape of this H_2_ p → o transition band is not discernible visually and the splitting of p → o transition is instead determined and analyzed by deconvolution of the broad band with multiple distribution functions.^19,20,63^ However, a very distinctively split band similar to the one observed in this work has also been observed for H_2_ which is adsorbed in two different adsorbents: (1) ultramicroporous carbon at *n*_H2_ value of 1.7 mmol g^–1^ and at temperatures of 5–77 K and (2) single-wall carbon nanotubes at 30% surface coverage and at 17 K, i.e., at similar conditions to those applied for sol–gel TiC-CDC.^17,19^

As the *T* increases, the *A*_tot_ value decreases which is explained by H_2_ desorbing and/or self-diffusing. The increase of Δ*E* value with increased *T* indicates that a still adsorbed fraction of H_2_ exhibits strong confinement through adsorption altering the p → o transition (Table 3). Therefore, even up to high *T*s of 80 K and under high applied H_2_ loadings, *n*_H2_ = 31 mmol g^–1^, high-energy adsorption sites, i.e., in ultramicropores, are able to effectively confine a detectable fraction of H_2_. The confined fraction is detected in a condition H_2_ is adsorbed beyond the monolayer, for example, at 60 K and under *n*_H2_ = 31 mmol g^–1^ where the surface coverage value is 160%. The increase in the *A*_free_/*A*_tot_ value (Table 3) with increased *n*_H2_ indicates that additional H_2_ adsorbs in larger pores and/or in lower energy adsorption sites, as smaller pores and higher energy adsorption sites are already populated, resulting in reduced anisotropic adsorption potential.

Similar shifts in the *E*_free_ value, as determined for sol–gel TiC-CDC, have been observed for H_2_ adsorbed in activated carbon, carbon nanotubes, and also shown by calculation (Table 3).^19−21^ Such a decrease in the band energy has been attributed to adjacent H_2_ molecules hindering each other and shifting the transition energies of different rotational states.^6,18−22^ The interaction of adjacent H_2_ molecules through the shift of the *E*_free_ value is most evident at *T* = 20 K and under the highest applied loading, *n*_H2_ = 31 mmol g^–1^. At these conditions, the highest surface coverage 294% is achieved and the strongest hindering effect of adjacent H_2_ molecules is yielded. With increased *T* the steric hindrance of adsorbed H_2_ molecules on each other is reduced, possibly due to the preferential desorption of freely rotating H_2_ molecules and creation of free adsorption sites.

No o → p conversion (i.e., the reverse transition to p → o) is detected for sol–gel TiC-CDC, even though the o → p conversion of H_2_ adsorbed in CDC synthesized from commercial TiC (com TiC-CDC) is reported in ref (22) at 10 K and *n*_H2_ = 2.0 mmol g^–1^. This difference could arise from the variations in the porous structure of the adsorbents used, where o-H_2_ can be preferentially adsorbed in some pores.^18^ Even though ultramicropores make up only 5% of all pores with 3 Å ≤ *w* ≤ 500 Å for sol–gel TiC-CDC, it could be a limiting factor toward the restriction of the o → p conversion to such a degree that it is still detectable in the experimental time frame afforded by instrument MARI. In contrast, for com TiC-CDC, 33% of all pores with 3 Å ≤ *w* ≤ 500 Å originate from ultramicropores.^8,10^ The larger percentage of ultramicropores in com TiC-CDC compared to that of sol–gel TiC-CDC supports the preferential adsorption of o-H_2_ and, thus, the porous structure of com TiC-CDC restricts the o → p conversion to such a degree that it is still detectable in the experimental time frame. All in all, the relatively low fraction of ultramicropores in sol–gel TiC-CDC is not able to restrict the H_2_ o → p conversion to the same degree and, thus, it is not possible to observe the H_2_ o → p conversion during the experimental neutron scattering time frame.

The restriction of the exothermic H_2_ o → p conversion would be of practical interest for cryo-adsorption or cryo-pressurized systems to prevent additional energy losses due to extra cooling requirements. Therefore, ultramicropores are essential for the effective storage of H_2_ for the preferential adsorption of o-H_2_ and limiting effect to the conversion of o-H_2_ to the para form, in addition to the effective confinement and self-diffusion restrictions of adsorbed H_2_.

At *n*_H2_ = 1.7 mmol g^–1^ and *T* ≥ 30 K, a fraction of H_2_ exhibits self-diffusion, although it is restricted and confined to jumps of 3.4 and 3.7 Å at 50 and 80 K, respectively, as was determined from EISF data. Two mechanisms for the adsorbed H_2_ motions are possible: (1) jumps between opposing pore walls and/or (2) jumps along the pore walls.

In the first case, H_2_ molecules exhibit oscillatory motions. Effectively, H_2_ molecules jump between potential energy minima created by the pore walls. Previous studies have shown that the H_2_ jumps with distances of 3.4 and 3.7 Å can take place in pores with a width of approximately 7 Å, i.e., in large ultramicropores or small supermicropores.^66^ In addition to the spatial limitation of the opposing ultramicropore walls, the neighboring adsorbed H_2_ molecules provide a steric barrier as, according to the pressure calculation, 21 and 13% of the monolayer is occupied by H_2_ at 50 and 80 K, respectively.

In the second case, H_2_ molecules diffuse along the pore walls jumping between strong adsorption sites located on the graphene surface. According to refs (9,67), these adsorption sites are separated by distances ranging between 3 and 5 Å, a range that overlaps with the 3.4 and 3.7 Å H_2_ jumps determined from EISF analysis. Thus, the H_2_ jumps along the pore walls would be between strong adsorption sites on the graphene-like surface.

The restricted H_2_ jumps - both between opposing ultramicropore walls and along the pore walls - show the confining effect of specific adsorption sites in carbon materials. No longer self-diffusive jumps were detected at these H_2_ loading and temperature conditions based on ref (8) and a strongly confined adsorbed H_2_ phase is present at all measured temperature and H_2_ loading conditions, based to the p → o conversion. Thus, the H_2_ determined to perform the restricted 3.4 and 3.7 Å jumps is the most mobile fraction of H_2_ which is still strongly adsorbed within the porous structure and any H_2_ not confined in the large ultramicropores or small supermicropores is desorbed at such low H_2_ partial pressures, ≤20 mbar (Table S2).

Such a strong H_2_ confinement in the ultramicropores lays the foundation for the formation of high-density adsorbed H_2_ phase and which has been investigated by other groups before. For example, the density of H_2_ adsorbed in micropores has been investigated with gas adsorption and small-angle neutron scattering methods and has been reported to exceed the liquid H_2_ density (71 kg m^–3^) at room temperature and 207 bar.^4^ Another study applying small-angle neutron scattering and gas adsorption methods showed that the density of H_2_ adsorbed in ultramicropores exceeds the bulk-liquid H_2_ density at 77 K and at 0.5 bar.^68^ Even in mesoporous materials, the density of adsorbed H_2_ can be increased compared to the densities of bulk-liquid and bulk-solid H_2_.^69^ Namely, the density of adsorbed H_2_ monolayer in mesoporous silica was investigated with gas adsorption, INS, and molecular dynamic simulations and has been reported to be 202 kg m^–3^.^69^ The formation of a high-density adsorbed H_2_ phase is a crucial step toward the enhancement of H_2_ storage capacity of porous materials. For example, it has been calculated that an adsorbent containing 1 cm^3^ g^–1^ of pores with *w* = 9 Å would be able to store 8.13 wt % of H_2_ at room temperature and 207 bar, which could approach the U.S. Department of Energy system-based target.^4^

## 5 Conclusions

The interactions of H_2_ adsorbed in the high-energy adsorption sites of a porous sol–gel TiC-derived carbon (sol–gel TiC-CDC) with hierarchical porosity from ultramicropores to mesopores and with relatively defect-free nongraphitic carbon structure. For that, *in situ* quasi- and inelastic neutron scattering methods are applied in the temperature range from 20 to 80 K and under H_2_ loadings, *n*_H2_, in the range of 1.7–31 mmol g^–1^, applied at 77 K, yielding surface coverages of up to 294% at 20 K.

At all investigated H_2_ loading and temperature conditions a fraction of effectively translationally bound, i.e., solid-like, but free-to-rotate adsorbed H_2_ was present. At the lowest applied H_2_ loading, *n*_H2_ = 1.7 mmol g^–1^, and in the whole investigated *T* range of up to 80 K, H_2_ was rotationally confined based on the para to ortho (p → o) transition band. Restricted self-diffusion of H_2_ was determined at *T* ≥ 30 K and between adsorption sites with distances of 3.4 and 3.7 Å, at 50 and 80 K, respectively, based on the quasi-elastic broadening of the spectra and results of EISF analysis. These restricted jumps between or along the ultramicropore walls demonstrate the confining effects of specific adsorption sites present in the large ultramicropores and small supermicropores of carbon materials, which restrict H_2_ mobility, localize the H_2_ molecules, and confine H_2_ in a situation where H_2_ in weaker adsorption sites is completely desorbed.

Based on this, ultramicropores play a crucial role in effective H_2_ storage through their capability to adsorb H_2_ as a high-density phase and also their ability to restrict the self-diffusion of H_2_. The strong confinement of adsorbed H_2_ supports the future use of porous materials for cryo-adsorptive H_2_ storage applications by increasing the H_2_ storage capacity, optimizing the storage conditions, and increasing the energy efficiency.

## Supporting Information Available

Surface coverage and pore occupancy calculations (section 1), H_2_ para-ortho transition band fitting results (sections 2), quasi-elastic neutron scattering fitting results (section 3), H_2_ ortho-para transition band (sections 4) (PDF)
