# Supplementary material for: Restricted Dynamics and Para-Ortho Conversion of H2 Adsorbed in Micro- and Mesoporous Carbide-Derived Carbon: A Quasi- and Inelastic Neutron Scattering Study
Source: J Phys Chem C Nanomater Interfaces. 2025 Feb 25;129(9):4789–99. doi: 10.1021/acs.jpcc.4c08582 (PMC11891908; doi:10.1021/acs.jpcc.4c08582)
Supplement: Supplementary file 1 — jp4c08582_si_001.pdf [file jp4c08582_si_001.pdf]

Supplementary Information

The Restricted Dynamics and Para-Ortho Conversion of H_2 Adsorbed in Micro- and Mesoporous Carbide-Derived Carbon: a Quasi- and Inelastic Neutron Scattering Study

Miriam Koppel1, Rasmus Palm1, Riinu Härmas1, Mark Telling2, Manh Duc Le2, Tatiana Guidi2,3, Kenneth Tuul1, Maarja Paalo1, Enn Lust1*

1 Institute of Chemistry, University of Tartu, Ravila 14a, 50411 Tartu, Estonia

2 ISIS Neutron and Muon Facility, STFC Rutherford Appleton Laboratory, Chilton, Didcot OX11 0QX, United Kingdom

3 School of Science and Technology, Physics Division, University of Camerino, I-62032 Camerino, Italy

1. Surface coverage and pore occupancy calculations

The dosing of H_2 and the calculations of H_2 surface coverage and pore occupancy are presented in Ref. [1]. Here, the most important aspects are summarized.

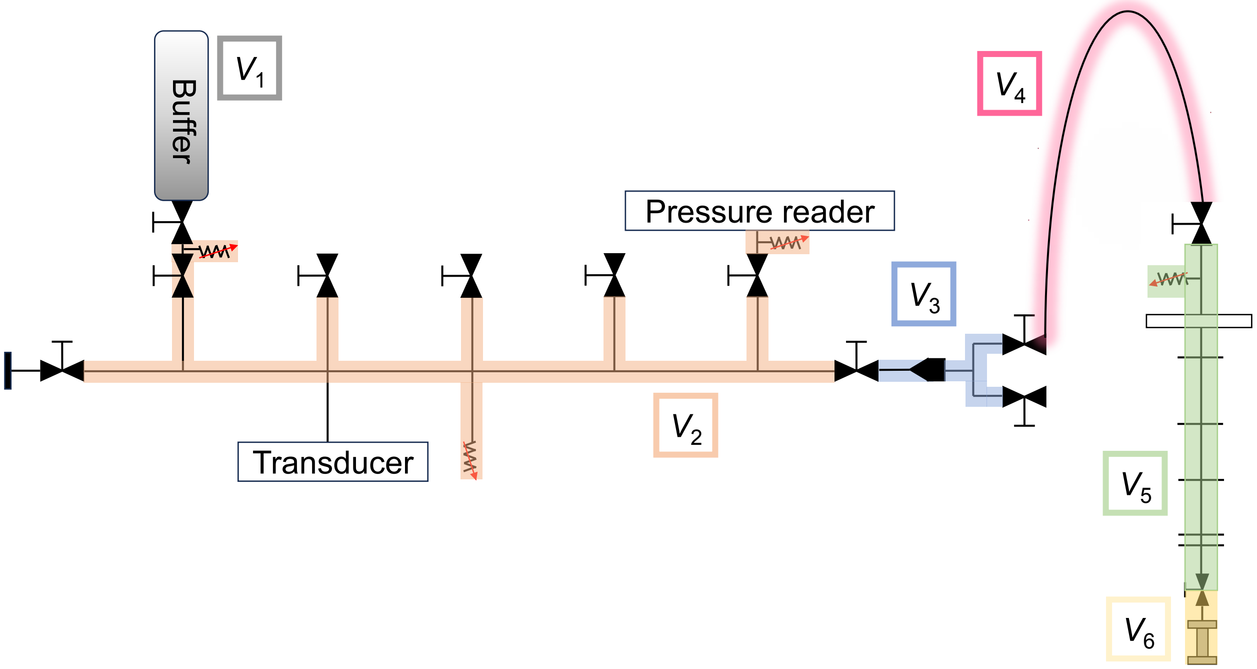

Figure S1. Schematics of the gas handling system used on IRIS and MARI spectrometers, where V_x marks volumes of the different parts of the system which can be closed off by valves. Reproduced from Ref. [1]. Available under a CC-BY 4.0 license. Copyright 2024 M. Koppel.

The exact H_2 loading pressures ($p_{H2,load}$) were calculated before loading the sample with H_2 to achieve identical surface coverage for both experiments on IRIS and on MARI. The gas handling system (Figure S1), sample cell and the amount of carbon adsorbent used for both experiments were slightly different. The volumes, V_x, of different components of the gas handling systems were calibrated with He before the experiments and can be seen in Table S1. Therefore, the $p_{H2,load}$ values were also different. The exact $p_{H2,load}$ values were for IRIS 14.9 mbar, 249 mbar, and 980 mbar, and for MARI 13.5 mbar, 225 mbar, and 969 mbar.

Table S1. Calibrated volumes of different components of gas handling systems on MARI and on IRIS.

<table>
<tr><th>Component</th><th>Component volume (MARI) / cm^3</th><th>Component volume (IRIS) / cm^3</th></tr>
<tr><td>V_1</td><td>491.2</td><td>499.8</td></tr>
<tr><td>V_2</td><td rowspan="2">41.5</td><td>33.8</td></tr>
<tr><td>V_3</td><td>5.2</td></tr>
<tr><td>V_4</td><td>24.1</td><td>14.9</td></tr>
<tr><td>V_5</td><td>3.7</td><td>11.2</td></tr>
<tr><td>V_6</td><td>16.2</td><td>10.7</td></tr>
</table>

The volumes of components V_1, V_2, V_3, V_4, V_5, and V_6 in Figure S1 can be seen in Table S1. For H_2 dosing, at first the volumes V_1 and V_2 in Figure S1 were opened and dosed with H_2 at 77 K. After that, the rest of the volumes (V_3, V_4, V_5, and V_6) were opened and the adsorption equilibrium was established for 1 h. The value on the pressure reader was noted as $p_{H2,load}$. Thereafter, V_1 was closed and the noted $p_{H2,load}$ values were used afterwards for the surface coverage calculations. After that, the temperature was lowered from 77 K to 10 K on IRIS and from 77 K to 20 K on MARI, and the neutron scattering measurement was started.

The H_2 pressures in the sample cell were monitored for both experiments on MARI and IRIS and can be seen in Table S2. These H_2 pressures were thereafter used to calculate the H_2 surface coverage and pore volume occupancy at each $p_{H2,load}$ and temperature condition. In the following, only the conditions studied in this publication will be shown, i.e., data measured with MARI at all the $p_{H2,load}$ values and at temperatures 20 K, 40 K, 60 K and 80 K and data measured with IRIS $p_{H2,load}$ = 14.9 mbar and at temperatures 50 K and 80 K. All of the data is brought in detail in Ref. [1].

Table S2. H_2 pressure readings in the closed sample cell in bar.

$p_{H2,load}$ / mbar	14.9	13.5	225	969
Instrument	IRIS	MARI	MARI	MARI
T=20 K	-*	0.0003	0.0002	0.0003
T=40 K	-*	0.0003	0.0003	0.12
T=50 K	0.0004	-*	0.008	0.30
T=60 K	-*	0.0005	0.05	0.53
T=80 K	0.02	0.020	0.27	1.1**

*Neutron scattering was not measured, therefore no pressure readings were obtained.

** High-pressure transducer with lower accuracy was used.

To calculate the amount of adsorbed H_2 (n_{ads}), Sips equation (Eq. S1) was applied [2].

$$n_{\mathrm{ads}} = n_{\mathrm{ads}}^{\mathrm{max}} \frac{(Kp)^{1/n}}{1 + (Kp)^{1/n}} \tag{S1}$$

where n_{ads} is the amount of adsorbed gas, $n_{\mathrm{ads}}^{\mathrm{max}}$ is the amount of adsorbed gas at full surface coverage, K is equilibrium coefficient, p is the adsorbate's partial pressure and n is heterogeneity coefficient.

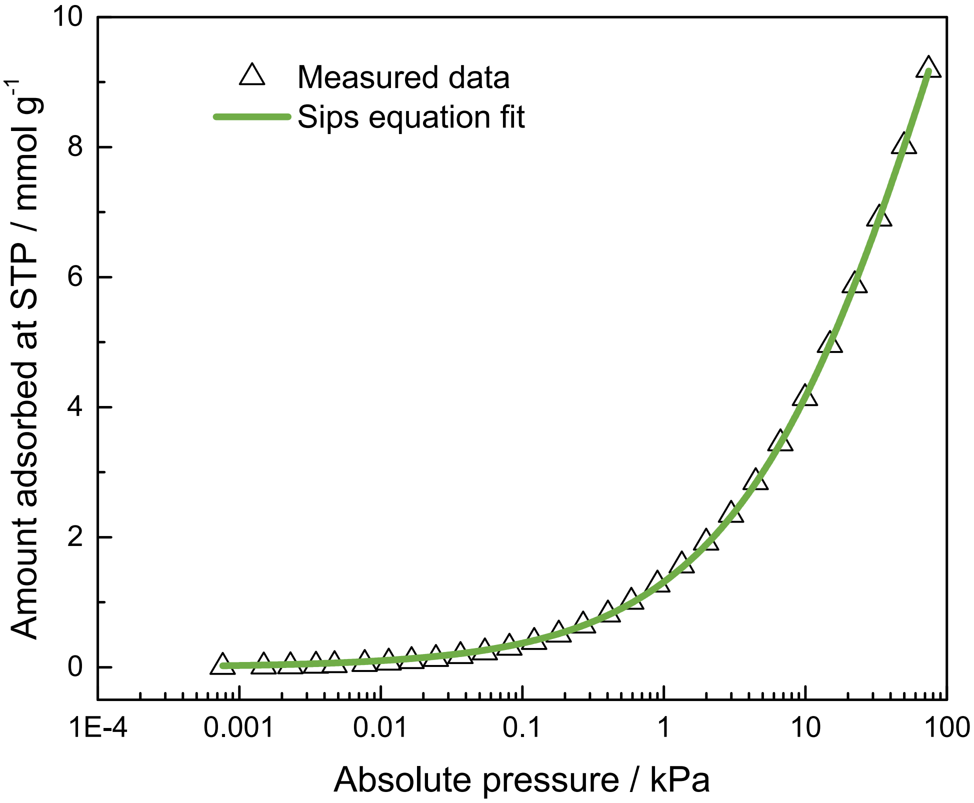

Figure S2. H_2 isotherm (black triangles) fitted with Sips equation (green line). Reproduced from Ref. [1]. Available under a CC-BY 4.0 license. Copyright 2024 M.Koppel.

H_2 adsorption isotherms measured at 77 K for sol-gel TiC-CDC was fitted with Sips equation (Figure S2) and the fitting results are in Table S3.

Table S3. Results of sol-gel TiC-CDC H_2 isotherm measured at 77 K and fitted with Sips equation.

n_{ads}^{max} / mmol g^{-1}	K / bar^{-1}	n / unitless
19.39	0.80	1.76

n_{ads}^{max} - amount of adsorbed gas at full surface coverage
K - equilibrium coefficient
n - heterogeneity parameter

The calculations were identical to the calculations in the supplementary information of Ref. [1,3]. The total amount of H_2 per 1 g of sol-gel TiC-CDC in the closed sample cell denoted as n_{H2} are

1.7 mmol g^{-1} (corresponding to $p_{H2,load}$ = 14.9 mbar on IRIS and $p_{H2,load}$ = 13.5 mbar on MARI), 10 mmol g^{-1} (corresponding to $p_{H2,load}$ = 225 mbar on MARI), and 31 mmol g^{-1} (corresponding to $p_{H2,load}$ = 969 mbar on MARI), respectively. The percentage of sol-gel TiC-CDC surface covered with H_2 is in Table S4 and calculated on the consideration that the surface area, S_{DFT}, is 1560 m^2 g^{-1} and that the cross-section of H_2 is $1.08 \cdot 10^{-19}$ m^2. A percentage value over 100% indicates that some of the H_2 is adsorbed in subsequent layers in addition to the adsorbed H_2 present in the monolayer. Pore occupancies of sol-gel TiC-CDC with H_2 by volume are in Table S5 and calculated on the consideration that the pore volume, V_{DFT}, is 2.40 cm^3 g^{-1} and that the density of adsorbed H_2 is equal to the density of liquid H_2.

Table S4. The surface of sol-gel TiC-CDC covered with H_2 in %, considering that the surface area, S_{DFT}, is 1560 m^2 g^{-1} and H_2 cross-section is $1.08 \cdot 10^{-19}$ m^2.

n_{H2} / mmol g^{-1}	1.7		10	31
Instrument	IRIS	MARI	MARI	MARI
T=20 K	-*	30	133	294
T=40 K	-*	30	133	255
T=50 K	21	-*	131	221
T=60 K	-*	30	121	161
T=80 K	13	25	76	-**

*Neutron scattering was not measured and, therefore, no pressure reading was obtained and no surface coverage was calculated.

**This value could not be calculated precisely as the pressure transducer was changed to the high-pressure one for which the free volumes were not known.

Table S5. Occupancy of sol-gel TiC-CDC pores with H_2 in % based on the consideration that the pore volume, V_{DFT}, is 2.40 cm^3 g^{-1} and that the density of adsorbed H_2 is equal to the density of liquid H_2.

n_{H2} / mmol g^{-1}	1.7		10	31
Instrument	IRIS	MARI	MARI	MARI
T=20 K	-*	2	11	24
T=40 K	-*	2	11	21
T=50 K	1	-*	11	17
T=60 K	-*	2	10	13
T=80 K	1	2	6	-**

*Neutron scattering was not measured and, therefore, no pressure reading was obtained and no pore occupancy was calculated.

**This value could not be calculated precisely as the pressure transducer was changed to the high-pressure one for which the free volumes were not known.

At 20 K and at n_{H2} = 1.7 mmol g^{-1}, ~2% of the total pore volume is occupied with H_2 assuming that the density of adsorbed H_2 is equal to that of liquid H_2. Surface coverage calculations show that at 20 K and at n_{H2} = 1.7 mmol g^{-1}, ~30% of the monolayer is filled. As ultramicropores make up ~5% of the total pore volume and the smallest pores are filled first, at n_{H2} = 1.7 mmol g^{-1}, H_2 occupies mostly ultramicropores which provide the strongest adsorption sites (the volume of ultramicropores and micropores of sol-gel TiC-CDC is 0.11 cm^3 g^{-1} and 0.52 cm^3 g^{-1}, respectively.)

At 20 K and at n_{H2} = 10 mmol g^{-1} and n_{H2} = 31 mmol g^{-1}, ~11% and ~24% of the total pore volume is occupied with H_2, respectively. Micropores make up ~30% of the total pore volume, meaning that at n_{H2} = 10 mmol g^{-1}, H_2 occupies most of the ultramicropores and some of the micro- and mesopores. At n_{H2} = 31 mmol g^{-1}, H_2 occupies almost all the micropores and some of the mesopores. The surface coverage calculations suggest that at 20 K and at n_{H2} = 10 mmol g^{-1} and n_{H2} = 31 mmol g^{-1}, ~130% and ~290% of the monolayer is filled, respectively. Thus, suggesting that at these conditions some of the adsorbed H_2 is in subsequent layers in addition to

the H_2 monolayer. This is further evidence that H_2 is adsorbed in both micro- and mesopores at 20 K.

2. Neutron energy loss region - para-ortho transition band

Para-ortho (p→o) transition band fitting results are in Figure S3 and in Table S6. A linear background is included in all the peaks. The reduced data is collated over all scattering vector, *Q*, values into one *Q* group. The H_2 p→o transition band is fitted with three Lorentzian functions. The H_2 p→o transition has shown to be split into two components of ~2:1 ratio [4–7]. Therefore, the intensity ratio of the lowest and highest energy bands ~2:1 was fixed throughout the fitting to get a stable fit.

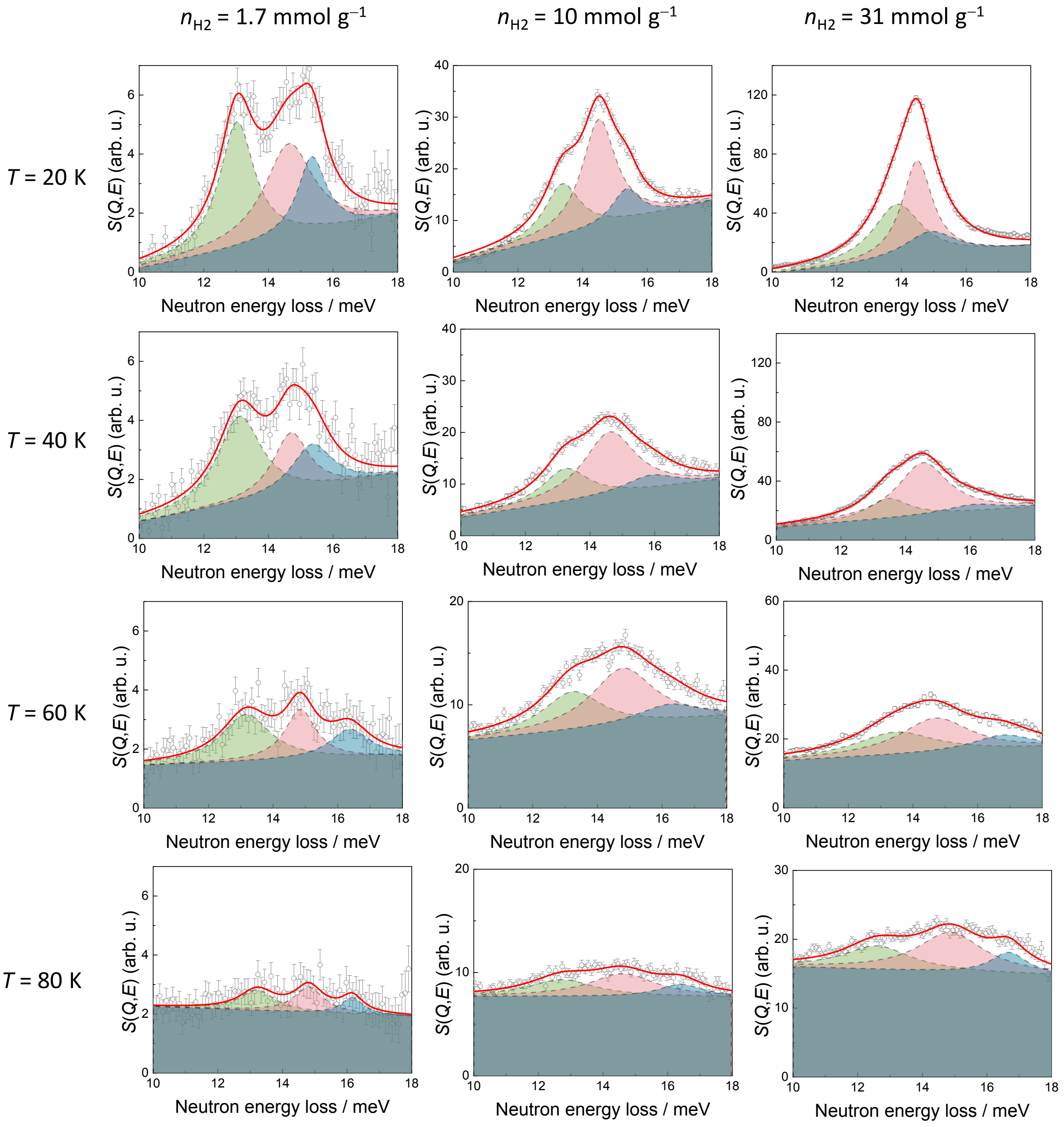

Figure S3. Fitting the H_2 p⟶o transitions peaks with three Lorentzian functions (background has not been subtracted).

At the energy transfer range 12–17 meV, the broad band corresponding to the H_2 p⟶o rotational transfer, J=0⟶1, is detected (Figure S3). p⟶o transition bands are visible for all studied n_{H2} values and for temperatures up to 80 K. As the temperature increases, the area of the band decreases (Table S6) because of the decrease in p-H_2 concentration and decrease in the level of surface

coverage and pore volume occupancy with H_2 (Table S4 and S5). With increased n_{H2}, the total area of the band increases as additional H_2 is adsorbed in lower energy adsorption centers and in subsequent layers (Table S6).

Table S6. Para-ortho rotational transition peak fitting results (error is below each parameter, parameters of each type of peaks are shaded with different colors).

n_{H2}	1.7 mmol g^{-1}				10 mmol g^{-1}				31 mmol g^{-1}			
T	20 K	40 K	60 K	80 K	20 K	40 K	60 K	80 K	20 K	40 K	60 K	80 K
x_c	13.02	13.11	13.16	13.22	13.37	13.24	13.23	12.73	13.59	13.46	13.47	12.60
dx_c	0.04	0.10	0.13	0.20	0.08	0.15	0.31	0.38	0.18	0.13	0.71	0.23
w	1.31	1.67	1.85	1.31	1.34	1.76	2.28	2.5	1.68	1.58	3.04	2.5
dw	0.16	0.33	0.53	0.76	0.19	0.43	0.84	1.28	0.31	0.37	1.11	0.92
A	8.86	7.82	4.66	1.42	21.58	18.08	13.62	6.41	58.51	33.97	32.18	13.51
dA	1.50	2.02	1.67	0.87	5.88	10.24	13.04	3.61	28.19	21.32	19.65	8.17
x_c	14.63	14.70	14.84	14.81	14.51	14.61	14.79	14.61	14.47	14.54	14.70	14.87
dx_c	0.33	0.10	0.08	0.14	0.04	0.11	0.19	0.22	0.03	0.06	0.34	0.13
w	1.86	1.38	1.17	1.09	1.28	2.02	2.40	2.5	1.44	1.94	2.68	2.5
dw	0.51	0.64	0.41	0.63	0.25	0.64	1.63	1.59	0.10	0.33	1.24	0.82
A	9.38	3.77	3.15	1.48	42.18	38.96	21.22	8.77	217.64	109.82	43.14	22.66
dA	7.07	3.15	1.31	0.84	13.54	22.28	28.43	5.62	32.61	35.28	13.68	9.88
x_c	15.34	15.35	16.37	16.16	15.35	15.87	16.23	16.41	16.07	16.10	16.79	16.68
dx_c	0.15	0.32	0.18	0.17	0.14	0.36	0.96	0.24	0.81	0.36	0.21	0.13
w	1.09	1.57	1.51	0.81	1.09	2.03	2.50	1.67	4.36	2.14	2.35	1.37
dw	0.59	0.73	0.68	0.64	0.36	0.91	1.96	1.10	0.63	0.76	0.76	0.63
A	4.43	3.91	2.33	0.71	10.79	9.04	6.81	3.20	29.26	16.99	16.09	6.76
dA	5.62	3.54	1.26	0.57	6.29	11.70	4.75	2.78	14.76	14.29	8.91	3.81

x_c – peak centre in meV
w – FWHM of the peak in meV
A – area of the peak in arbitrary units

3. Quasi-elastic neutron scattering (QENS) fitting results

The IRIS QENS spectra at n_{H2} = 1.7 mmol g^{-1} are fitted with the model containing one Lorentzian function (L1) accounting for one quasi-elastic broadening (Figure S4).

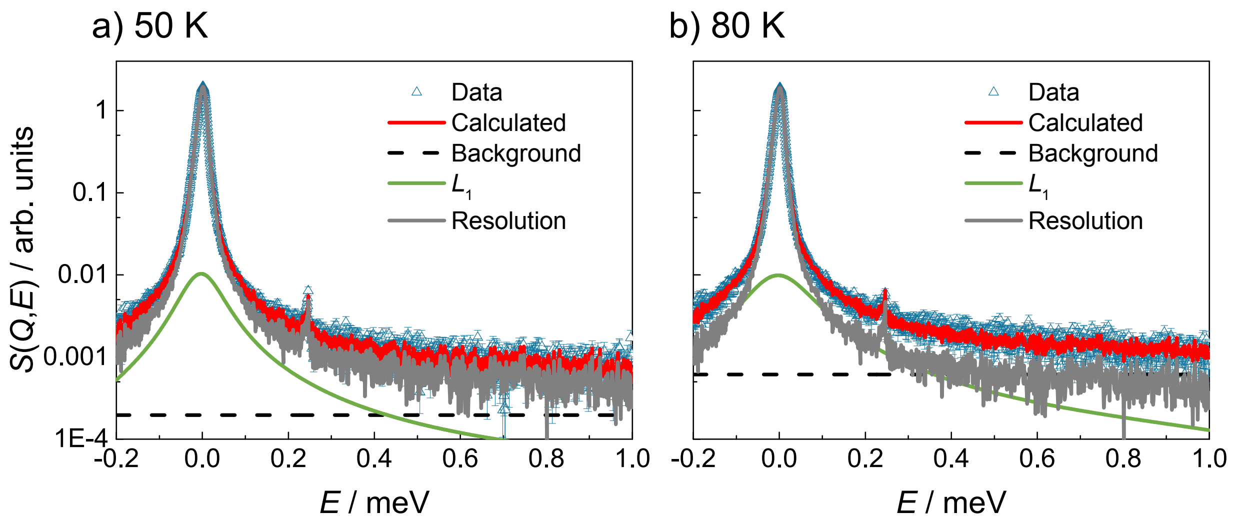

Figure S4. IRIS fitting results at n_{H2} = 1.7 mmol g^{-1} at a) 50 K and b) 80 K (all Q-values are summed). Reproduced from Ref. [1]. Available under a CC-BY 4.0 license. Copyright 2024 M.Koppel.

For EISF analysis, the Q-s were collated into five Q-groups. Example fits at Q = 1.0 Å^{-1} and Q = 1.6 Å^{-1} can be seen in Figure S5a and Figure S5b, respectively.

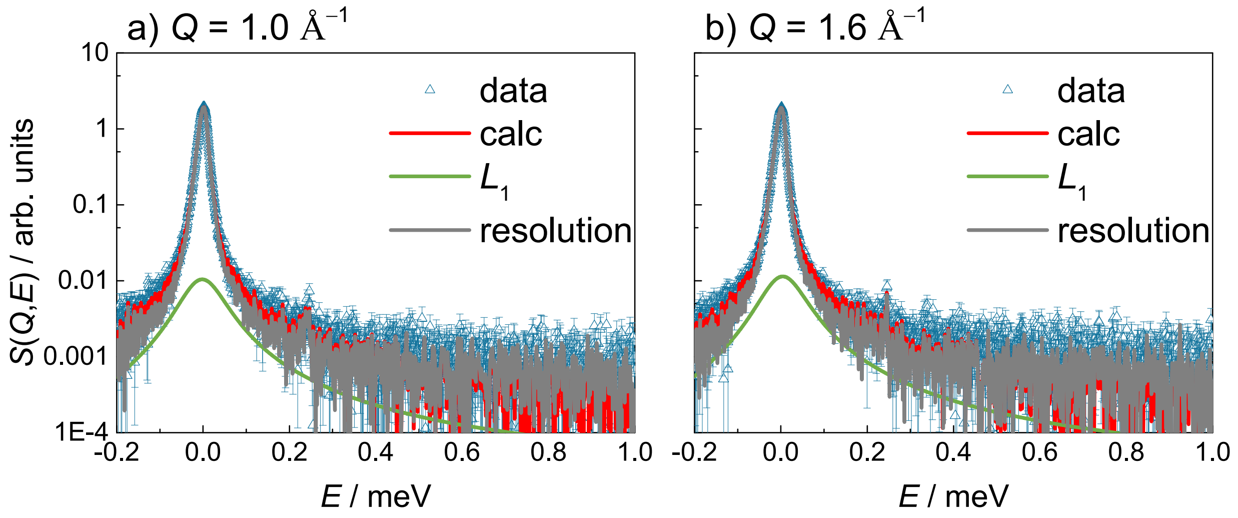

Figure S5. IRIS fitting results at $n_{H2,load}$ = 1.7 mmol g^{-1} at and at 50 K at a) Q = 1.0 Å^{-1} and b) Q = 1.6 Å^{-1}. Reproduced from Ref. [1]. Available under a CC-BY 4.0 license. Copyright 2024 M.Koppel.

The H_2 motions are analyzed by fitting experimentally determined EISF values with theoretical models. Integrated intensity of the elastic region from quasi-elastic neutron scattering measurement is plotted on Figure S6.

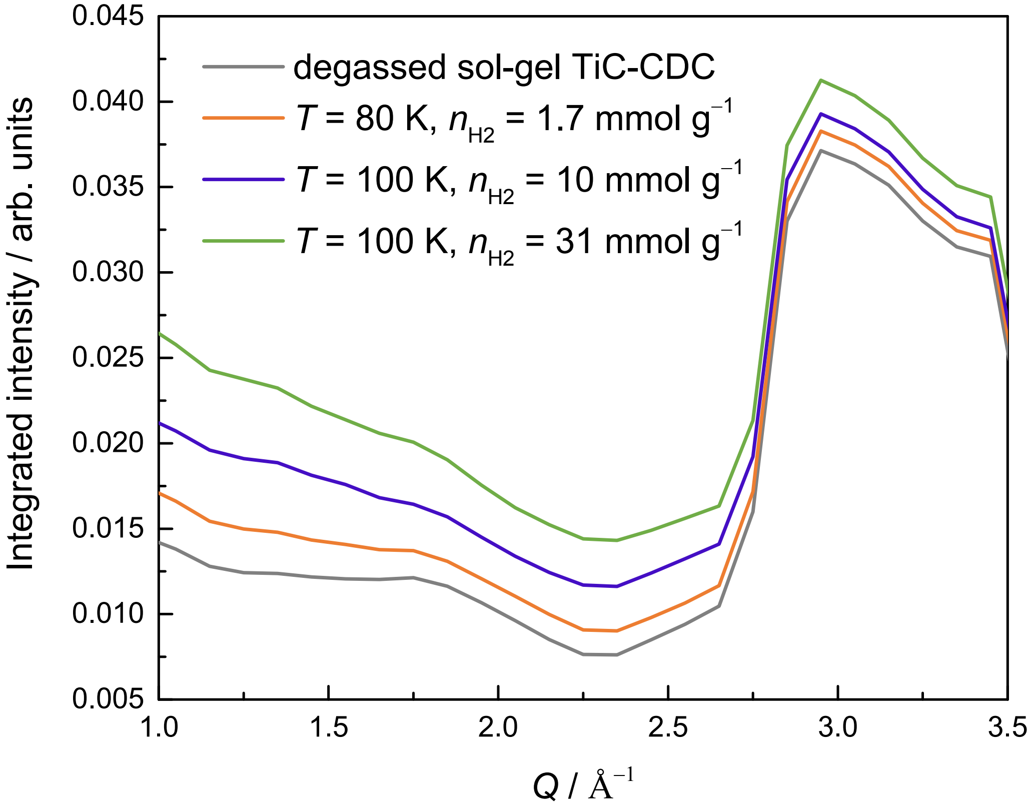

Figure S6. Integrated intensity of the elastic region from quasi-elastic neutron scattering measurement. Reproduced from Ref. [1]. Available under a CC-BY 4.0 license. Copyright 2024 M.Koppel.

The integrated intensity signal decreases up to $Q \approx 2.5$ Å^{-1} (Figure S6). Therefore, the distinctive shape of the EISF vs Q dependence, which start increasing from $Q \approx 1.3$ Å^{-1} at 50 K and $Q \approx 1$ Å^{-1} at 80 K, does not arise from the structure of sol-gel TiC-CDC but from the motions of adsorbed H_2 (Figure S7).

In this work, three different EISF models are applied:

1) Rotational jumps between two equidistant sites on a circle with diameter d [8].

$$A_{0,2site}(Q) = \frac{1}{2}(1 + j_0(Qd)) \tag{S2}$$

where j_0 is the zero order spherical Bessel function of the first kind.

2) Continuous rotational diffusion on the surface of a sphere with diameter d [8].

$$A_{0,\mathrm{sph}}(Q) = j_0^2(0.5Qd) \tag{S3}$$

3) Continuous rotational diffusion within the volume of a sphere with diameter d [8].

$$A_{0,\mathrm{vol}}(Q) = \left[\frac{3\,j_1(0.5Qd)}{0.5Qd}\right]^2 \tag{S4}$$

where j_1 is the first order spherical Bessel function of the first kind.

The fitting results with different experimental models can be seen in Figure S7 and Table S7.

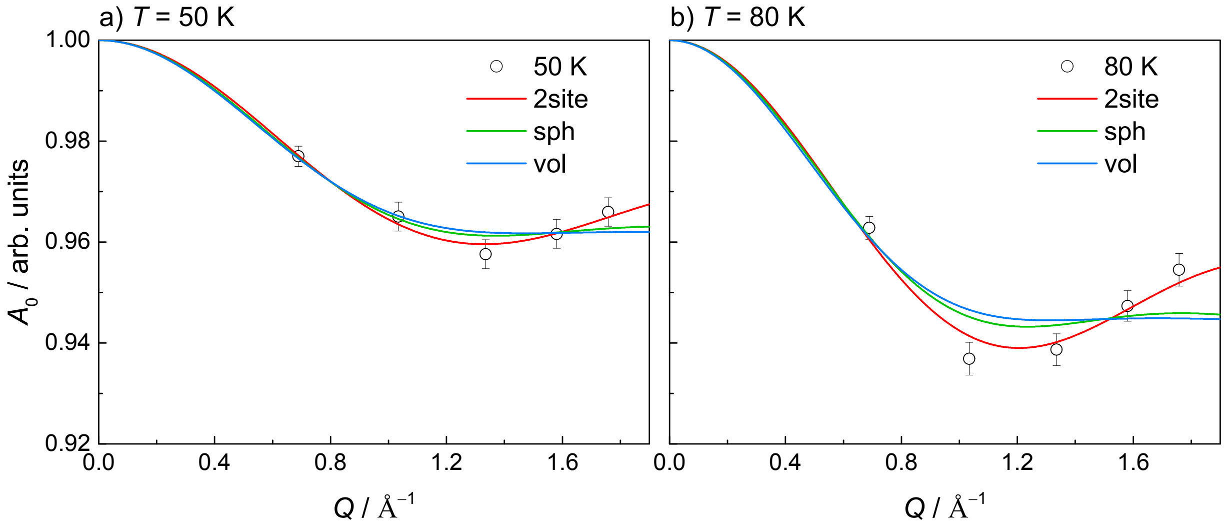

Figure S7. Fitting experimental EISF values with theoretical models describing rotational jumps between two equidistant sites on a circle with diameter d (2site, Eq. S2), continuous rotational diffusion on the surface of a sphere with diameter d (sph, Eq. S3) and continuous rotational diffusion within the volume of a sphere with diameter d (vol, Eq. S4) at a) 50 K and b) 80 K. Reproduced from Ref. [1]. Available under a CC-BY 4.0 license. Copyright 2024 M.Koppel.

Table S7. Results of fitting experimental EISF values with models, where $A_{0,\mathrm{2site}}$ denotes model describing rotational jumps between two equidistant sites on a circle with diameter d, $A_{0,\mathrm{sph}}$ denotes

model describing continuous rotational diffusion on the surface of a sphere with diameter *d*, and $A_{0,vol}$ denotes model describing and continuous rotational diffusion within the volume of a sphere with diameter *d*.

T / K	Parameter	$A_{0,2site}$	$A_{0,sph}$	$A_{0,vol}$
50 K	*d* / Å	3.4±0.1	4.6±0.3	6.1±0.5
	p_m	0.07±0.01	0.04±0.01	0.04±0.01
	R^2	0.98	0.92	0.89
80 K	*d* / Å	3.7±0.1	5.1±0.6	6.8±1.1
	p_m	0.10±0.01	0.06±0.01	0.06±0.01
	R^2	0.94	0.73	0.64

d - diameter of the confining volume
p_m - mobile fraction of adsorbed H_2

The best fits are achieved when the experimental EISF values are fitted with the model describing rotational jumps between two equidistant sites on a circle with diameter *d* ($A_{0,2site}$, Eq. 2) indicated by the R^2 values which are the closest to 1. Therefore, based on these results, the mobile fraction of H_2 adsorbed in sol-gel TiC-CDC is performing rotational jumps between two equidistant sites on a circle with a diameter of 3.4 Å and 3.7 Å at 50 K and 80 K, respectively.

4. Neutron energy gain region - H_2 ortho-para transition band

To analyze the reverse transition of p→o, ortho-para (o→p) transition of H_2 adsorbed in sol-gel TiC-CDC, the $S(Q,E)$ values are plotted in energy transfer window of −18 meV to −5 meV (Figure S8). The o→p transition of adsorbed H_2 has been observed around −14.7 meV [4]. However, no o→p transition of H_2 adsorbed in sol-gel TiC-CDC is seen.

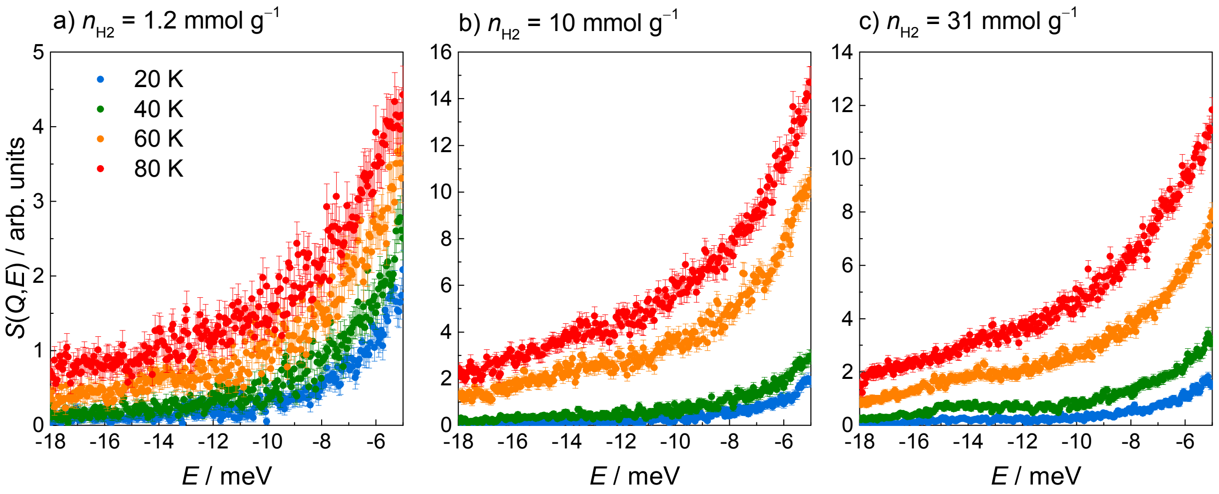

Figure S8. The neutron energy gain region of the spectra for H_2 adsorbed in sol-gel TiC-CDC.

5. References

[1] Koppel M, Palm R, Härmas R, Telling M, Le MD, Guidi T, Tuul K, Paalo M, Kalder L, Jagiello J. et al. Disentangling the self-diffusional dynamics of H2 adsorbed in micro- and mesoporous carbide-derived carbon by wide temporal range quasi-elastic neutron scattering. Carbon 2024;219:118799. https://doi.org/10.1016/j.carbon.2024.118799.

[2] Do DD. Adsorption Analysis: Equilibria and Kinetics. United Kingdom: Imperial College Press, London; 1998.

[3] Koppel M, Palm R, Härmas R, Russina M, Grzimek V, Jagiello J, Paalo M, Kurig H, Månsson M, Oll O et al. Pore wall corrugation effect on the dynamics of adsorbed H2 studied by in situ quasi-elastic neutron scattering: Observation of two timescaled diffusion. Carbon 2022;197:359–67. https://doi.org/10.1016/j.carbon.2022.06.061.

[4] Terry LR, Rols S, Tian M, Silva I da, Bending SJ, Ting VP. Manipulation of the crystalline phase diagram of hydrogen through nanoscale confinement effects in porous carbons. Nanoscale 2022;14:7250–61. https://doi.org/10.1039/D2NR00587E.

[5] Georgiev PA, Ross DK, Albers P, Ramirez-Cuesta AJ. The rotational and translational dynamics of molecular hydrogen physisorbed in activated carbon: A direct probe of microporosity and hydrogen storage performance. Carbon 2006;44:2724–38.

[6] Fukutani K, Sugimoto T. Physisorption and ortho–para conversion of molecular hydrogen on solid surfaces. Progress in Surface Science 2013;88:279–348. https://doi.org/10.1016/j.progsurf.2013.09.001.

[7] Fernandez-Alonso F, Bermejo FJ, Cabrillo C, Loutfy RO, Leon V, Saboungi ML. Nature of the bound states of molecular hydrogen in carbon nanohorns. Phys Rev Lett 2007;98:215503. https://doi.org/10.1103/PhysRevLett.98.215503.

[8] Bee M. Quasielastic neutron scattering. United Kingdom: Adam Hilger; 1988.
